# Supplementary material for: Profiling of the Helicobacter pylori redox switch HP1021 regulon using a multi-omics approach
Source: Nat Commun. 2023 Oct 23;14:6715. doi: 10.1038/s41467-023-42364-6 (PMC10593804; doi:10.1038/s41467-023-42364-6)
Supplement: Supplementary file 1 — Supplementary Information [file 41467_2023_42364_MOESM1_ESM.pdf]

Supplementary Information

Profiling of the *Helicobacter pylori* redox switch HP1021 regulon using a multi-omics approach

Mateusz Noszka[1], Agnieszka Strzałka[2], Jakub Muraszko[1], Rafał Kolenda[3,4], Chen Meng[5], Christina Ludwig[5], Kerstin Stingl[6], Anna Zawilak-Pawlik[1*]

[1] Department of Microbiology, Hirszfeld Institute of Immunology and Experimental Therapy, Polish Academy of Sciences, 53-114 Wrocław, Poland

[2] Department of Molecular Microbiology, Faculty of Biotechnology, University of Wrocław, 50-383 Wrocław, Poland

[3] Department of Biochemistry and Molecular Biology, Wrocław University of Environmental and Life Sciences, 50-375 Wrocław, Poland

[4] Quadram Institute Biosciences, Norwich Research Park, NR4 7UQ Norwich, United Kingdom

[5] Bavarian Center for Biomolecular Mass Spectrometry (BayBioMS), Technical University of Munich (TUM), 85354 Freising, Germany

[6] Department of Biological Safety, National Reference Laboratory for Campylobacter, German Federal Institute for Risk Assessment, 12277 Berlin, Germany

[*] to whom correspondence should be addressed: Anna Pawlik, tel. +48 71 3709949, e-mail: anna.pawlik@hirszfeld.pl

This PDF file includes:

Supplementary Table 1 to 2

- Tab. S1: Strains, plasmids and protein used in this study.
- Tab. S2: Primers used in this study.

Supplementary Figure 1 to 12

- Fig. S1: Identification of HP1021 binding sites on *H. pylori* N6 genome by ChIP-seq.
- Fig. S2: HP1021 controls *vacA* expression.
- Fig. S3: The reproducibility of biological replicates in omics data.
- Fig. S4: Overview of the gene regulation mediated by HP1021 revealed by RNA-seq.
- Fig. S5: Overview of the protein level regulation mediated by HP1021 revealed by MS/LC-MS.
- Fig. S6: *H. pylori* N6 Clusters of Orthologous Groups (COG).
- Fig. S7: HP1021 controls *katA* and *kapA* expression.
- Fig. S8: HP1021 controls *rocF* expression.
- Fig. S9: Analysis of DNA uptake by *H. pylori* P12.
- Fig. S10: HP1021 controls tRNA expression.
- Fig. S11: HP1021 controlled glucose uptake via GluP transporter.
- Fig. S12: The model of glucose metabolism in *H. pylori* N6.

Supplementary References

Supplementary Table

Table S1: Strains, plasmids and protein used in this study.

Strain	Relevant features	Reference/source
E. coli DH5α	*supE44*, *hsdR17*, *recA1*, *endA1*, *gyrA1*, *gyrA96*, *thi-1*, *relA1*	[1]
E. coli BL21	F-, *ompT*, *hsdS (rB-, mB-)*, *gal*, *dcm*	GE Healthcare
H. pylori 26695	Parental strain	[2]
H. pylori N6	Parental strain	[3]
H. pylori N6 ΔHP1021	Δ*HP1021*::*aphA-3*; N6 with *HP1021* exchanged to *aphA-3* cassette	[4]
H. pylori N6 COM/HP1021	(Δ*HP1021*::*aphA-3*):Δ*HP1021-cat*); N6 Δ*HP1021* in which *aphA-3* was exchanged to *HP1021* and *cat* cassette	[4]
H. pylori P12 ΔHP1021	Δ*HP1021*::*aphA-3*; N6 with *HP1021* exchanged to *aphA-3* cassette	This study
H. pylori P12 COM/HP1021	(Δ*HP1021*::*aphA-3*):Δ*HP1021-cat*); N6 Δ*HP1021* in which *aphA-3* was exchanged to *HP1021* and *cat* cassette	This study
H. pylori N6 ΔgluP	Δ*gluP*::*aphA-3*; N6 whit *gluP* exchanged to *aphA-3* cassette	This study
H. pylori N6 COM/gluP	(Δ*gluP*::*aphA-3*):Δ*gluP*-*cat*); N6 Δ*gluP* in which *aphA-3* was exchanged to *HPgluP* and *cat* cassette	This study

Plasmid	Relevant features	Reference/source
pUC18	Cloning vector, Amp^R	Thermo Fisher Scientific
pUC18/ΔgluP	pUC18 derivative containing *gluP* flanking regions for allelic exchange of *gluP* for *aphA-3*	This study
pUC18/ COM/gluP	pUC18 derivative containing *gluP* flanking regions and *gluP* for allelic exchange of *aphA-3* for *gluP-cat*	This study
pTZ57R/TΔHP1021	pTZ57R/T derivative containing *HP1021* flanking regions for allelic exchange of *HP1021* for *aphA-3*	[5]
pUC18/COM/HP1021	pUC18 derivative containing *HP1021* flanking regions and *HP1021* for allelic exchange of *aphA-3* for *HP1021-cat*	[4]
pET28/StrepHP1021	pET28Strep derivative containing the *HP1021* gene amplified with primers P-3/P-4 and cloned between BamHI and XhoI sites	[4]
pori2	A pOC170 derivative containing *oriC2*	[6]

Recombinant protein	Relevant features	Reference/source
StrepHP1021	Recombinant, *H. pylori* HP1021 protein, Strep-tagged at N-terminus, purified from *E. coli*	[4]

Table S2: Primers used in this study.

Oligo name	Sequence (5' → 3')
P1	gtcgactctagaggatccccgtaaaggtgaagtgccttatc
P2	gggtataagcaagaagaaaac
P3	gttttcttcttgcttatacccgctagcctaaaacaattcatccagtaaaata
P4	ctcaataaaaaaggagaaggtttaattaaggatcctgactaactaggagga
P5	accttctcctttttattgag
P6	cgaattcgagctcggtacccgcaatgcgatttatccggtg
P7	ttaggagttttcttcttgcttatac
P8	agcaagaagaaaactcctaacggaatttacggaggataaa
P9	gttttcttcttgcttatacccttacgccccgccctgccact
P10	atgcaaaaaacttctaacactctg
P11	ttaacgctcttttgcttgcc
P12	tccccaagcaaagtcgtgag
P13	gctcaccacgagcggcgatttg
P14	gctttaaaatcttcatcaaattgg
P15	ctagcggattctctcaatgtcaa
P16	ggagtacggtcgcaagattaaa
P17	tcaagtcttcaaagcgttgccacac
P18	aatgaagcgttggctcgttcgctc
P19	gcctttgacccaataatgcc
P20	ccaataaaaccccagataaaccc
P21	gttttctaaatgcttttcttggtgg
P22	gggacttgtgtttttggttg
P23	tcagtcgacgctctttttaaggggctttg
P24	tttttgcataccttctccttttt
P25	gtgatgctgaccaatgctcc
P26	tttctagtctaaagtcgcaccc
P27	catcgcgcaaaccatctcgc
P28	gacggacaataaggggcgct
P29	ggagtaagaatagcttcgaatcgaaagcgtctctaaagaaaa
P30	cgggatcctcatgcgagatttaacctgt
P31	ggagtaagaatagcttcgaatctatatatttatagccttaatcg
P32	cggcttgtttgagcccccag
P33	FAM-ggagtaagaatagcttcgaat*
P34	catcgataggatatcctggg

* - FAM, 6-fluorescein amidite.

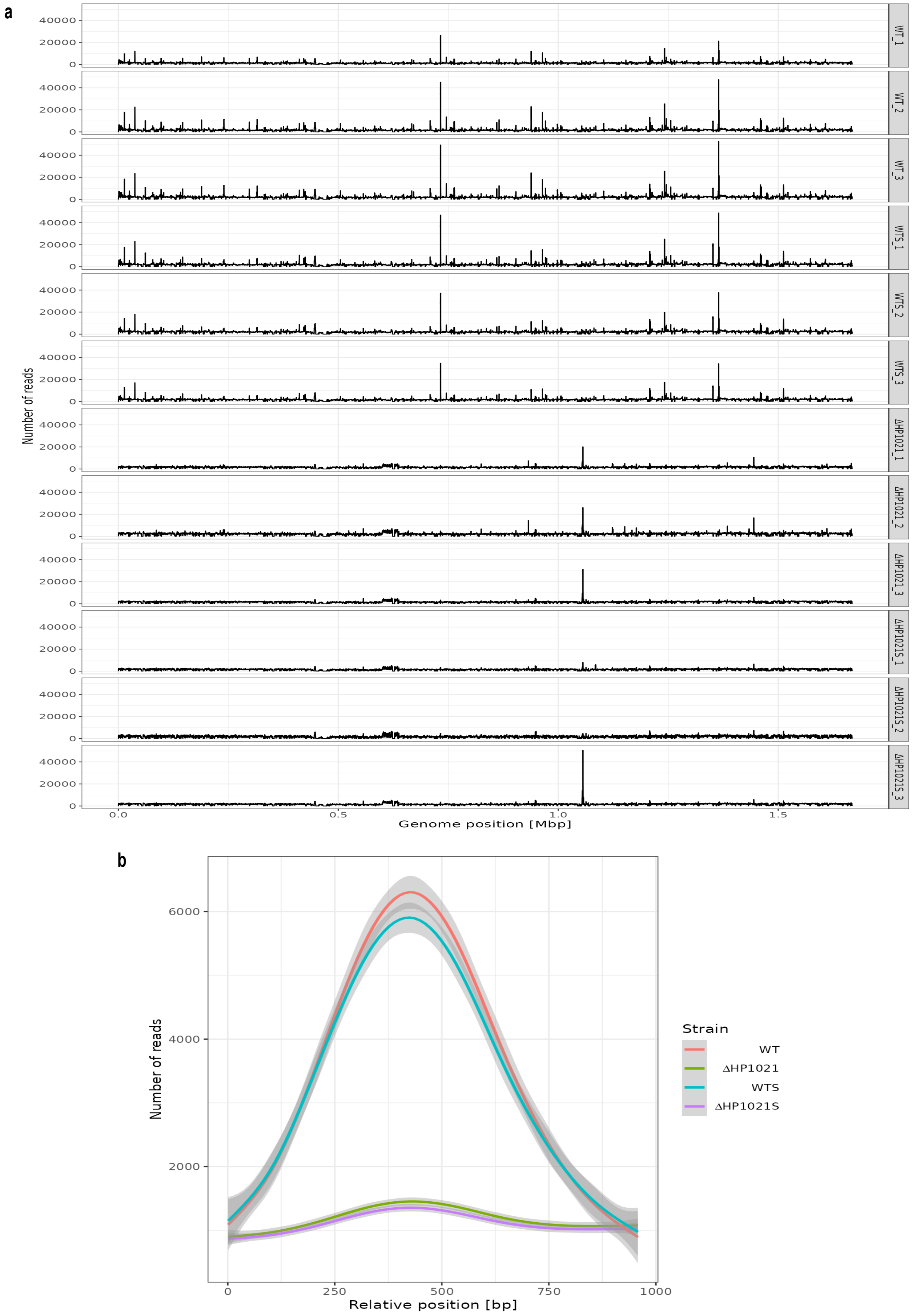

Fig. S1: Identification of HP1021 binding sites on *H. pylori* N6 genome by ChIP-seq. a ChIP-seq analysis of HP1021 binding to *H. pylori* N6 chromosome. The ChIP-seq reads for HP1021 in *H. pylori* N6 WT and ΔHP1021 mutant strains cultured under microaerobic and aerobic conditions (5% and 21% O2, respectively). Three independent analyses for each strain and condition are presented. **b** Comparison of HP1021-DNA interactions in the WT strain under microaerobic (red line) and aerobic (blue line) conditions with the control strain ΔHP1021 cultured under microaerobic (green line) and aerobic (purple line) conditions. The lines show mean values of reads (for 100 bp long regions) with 95% confidence intervals for 56 sites differentially bound by HP1021 protein according to edgeR analysis. For all sites, 1000 bp long fragments were extracted from the chromosome centred around the best position as determined by edgeR analysis.

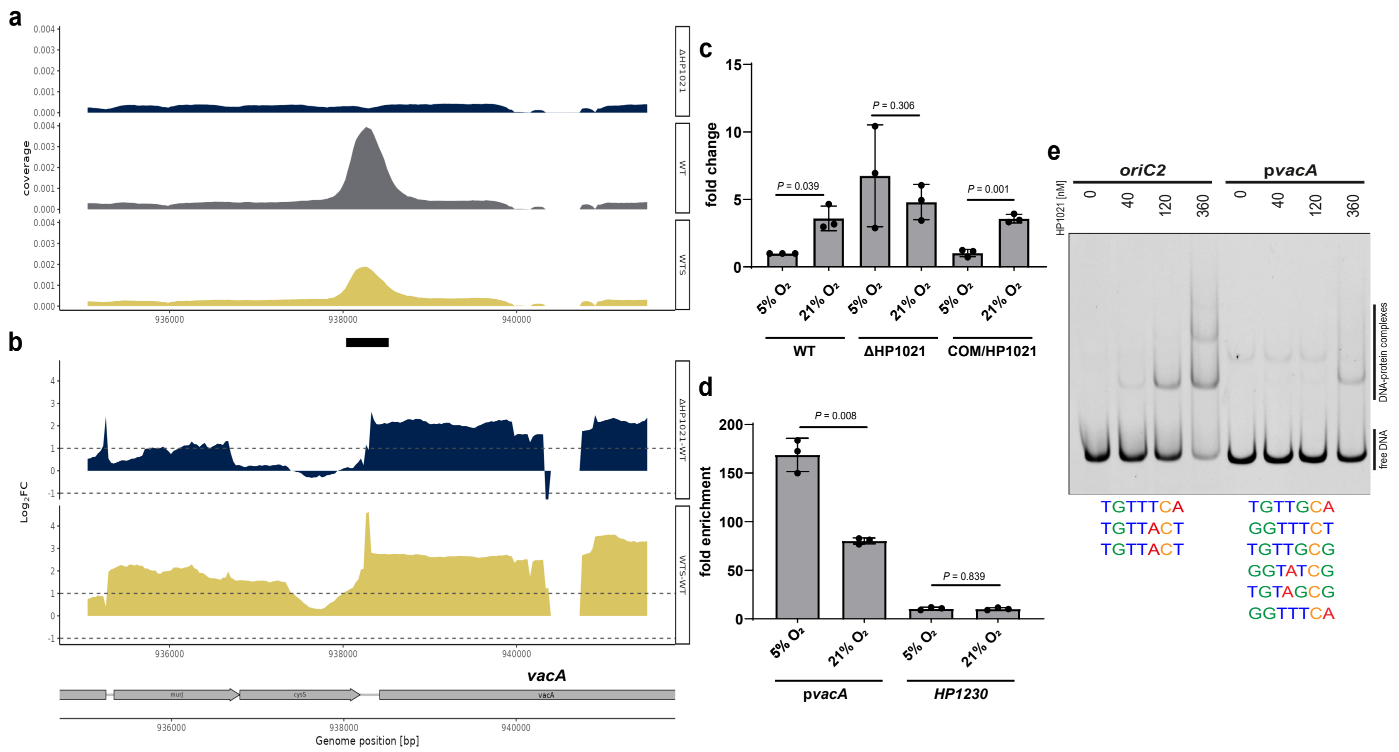

Fig. S2: HP1021 controls *vacA* expression. a ChIP-seq data profile of the *vacA* gene. Read counts were determined for *H. pylori* N6 WT, WTS and ΔHP1021 strains. The y-axis represents the coverage of the DNA reads, while the x-axis represents the position of the genome (in bps). The main peak of the binding site is marked with a thick black line under the x-axis. **b** RNA-seq data profile of *vacA* gene. The genomic locus for *H. pylori* N6 WT, WTS and ΔHP1021 strains with the WTS-WT and ΔHP1021-WT expression comparison; values above the black dashed lines indicate a change in the expression of $|\log_2 FC| \geq 1$; FDR ≤ 0.05. **c** RT-qPCR analysis of the transcription of *vacA* in *H. pylori* N6 cells cultured under microaerobic and aerobic conditions (5% and 21% O_2, respectively). The results are presented as the fold change compared to the WT strain. **d** ChIP fold enrichment of DNA fragment in *vacA* analyzed by ChIP-qPCR in *H. pylori* N6 cells cultured under microaerobic and aerobic atmosphere (5% and 21% O_2, respectively). The *HP1230* gene was used as a negative control not bound by HP1021. **e** EMSA analysis of HP1021 binding to the p*vacA* region *in vitro*. EMSA was performed using the FAM-labeled DNA fragments and recombinant Strep-tagged HP1021. The *oriC2* DNA fragment was used as a high-affinity control. The HP1021 boxes (putative in the promoter p*vacA* region and experimentally determined in the *oriC2* region) are shown below the gel image. Digital processing was applied equally across the entire image, including controls. The experiment was repeated twice with similar results. **c-d** Data are depicted as the mean values ± SD. Two-tailed Student's t-test determined the P value. n = 3 biologically independent experiments. Source data are provided with this paper.

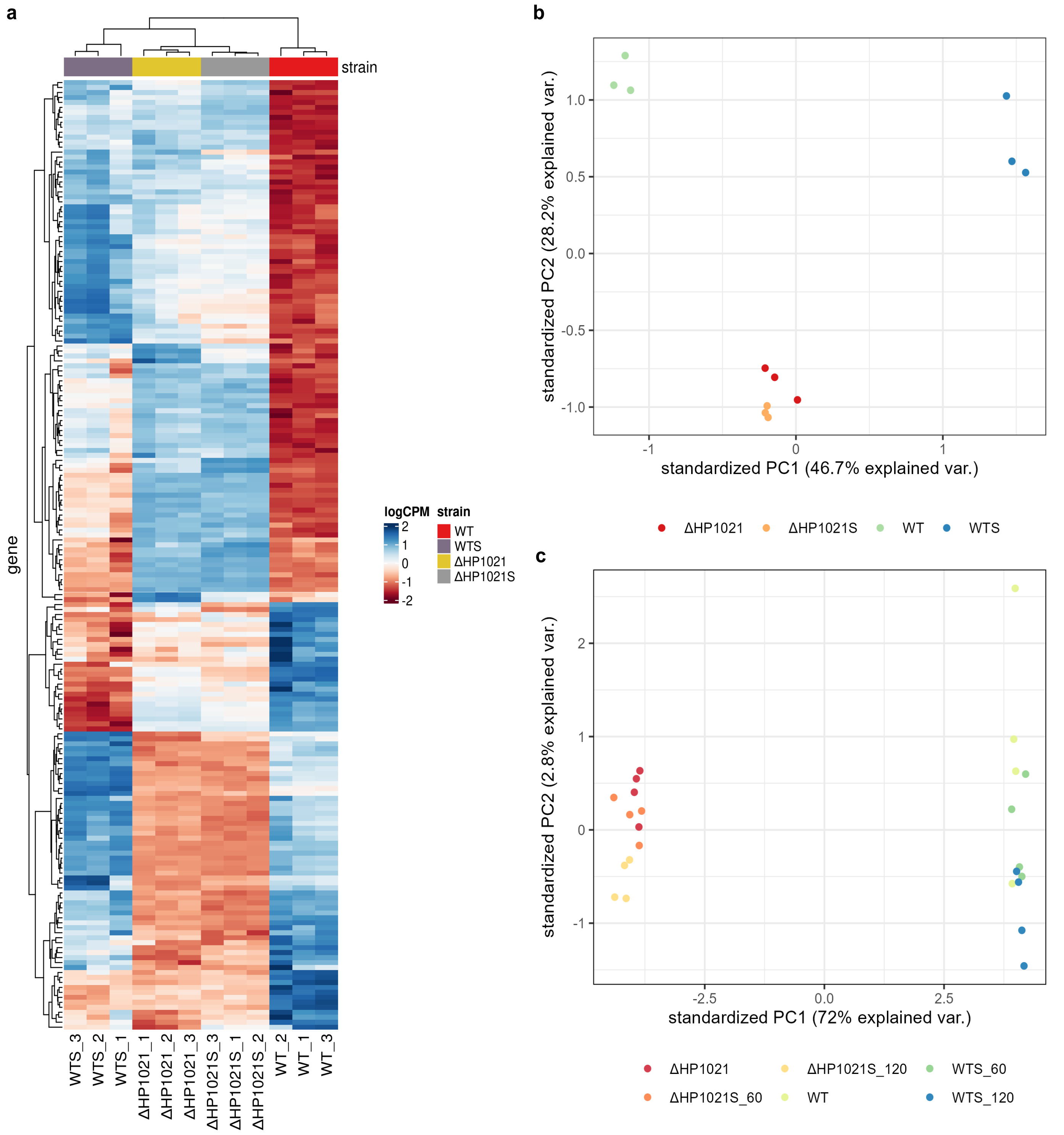

Fig. S3: The reproducibility of biological replicates in omics data. a Heat map of RNA-seq transcriptome. Analysis for 191 genes differentially transcribed ($|\log_2 FC| \geq 1$ and FDR ≤ 0.05) in *H. pylori* N6 ΔHP1021 mutant strain compared to the WT strain. Plotted values are log-Counts-Per-Million (logCPM) normalized expression values of the differentially expressed gene. Data are normalized for library size and scaled to the same mean (0) and standard deviation for each gene. **b** Principal component analysis (PCA) of the normalized RNA-seq CPM data of *H. pylori* N6 strains under microaerobic and aerobic conditions (5% and 21% O_2, respectively). **c** PCA of the normalized proteomics CPM data of *H. pylori* N6 strains under microaerobic conditions (WT, ΔHP1021) and in response to oxidative stress after 60 minutes (WTS_60, ΔHP1021S_60) and 120 minutes (WTS_120, ΔHP1021S_120).

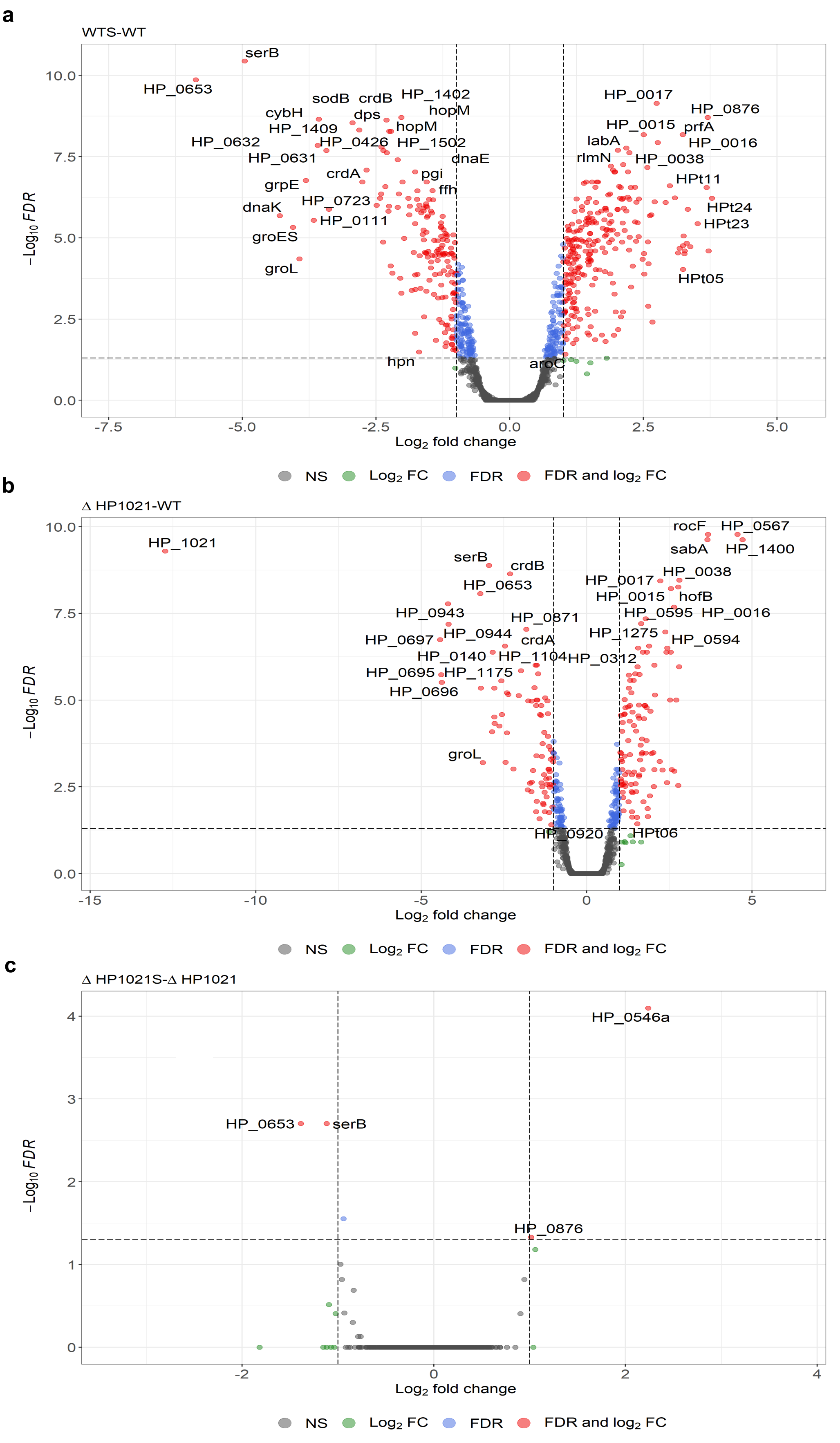

Fig. S4: Overview of the gene regulation mediated by HP1021 revealed by RNA-seq. a Volcano diagram of genes differently transcribed in the ΔHP1021 mutant strain compared to the wild-type (WT) strain (ΔHP1021-WT). **b** Volcano diagram of genes differently transcribed in the wild-type strain under oxidative stress (WTS) compared to the non-stressed wild-type strain (WTS-WT). **c** Volcano diagram of genes differently transcribed in the ΔHP1021 strain under oxidative stress (ΔHP1021S) compared to the non-stressed ΔHP1021 mutant (ΔHP1021S-ΔHP1021). **a-c** Three independent biological replicates were analyzed. Green dots correspond to genes with $|\log_2 FC| \geq 1$ and $FDR \geq 0.05$; blue dots correspond to genes with $|\log_2 FC| \leq 1$ and $FDR \leq 0.05$; red dots correspond to genes with $|\log_2 FC| \geq 1$ and $FDR \leq 0.05$; grey dots correspond to genes that were not significantly changed. NS, non significant. FDR, false discovery rate.

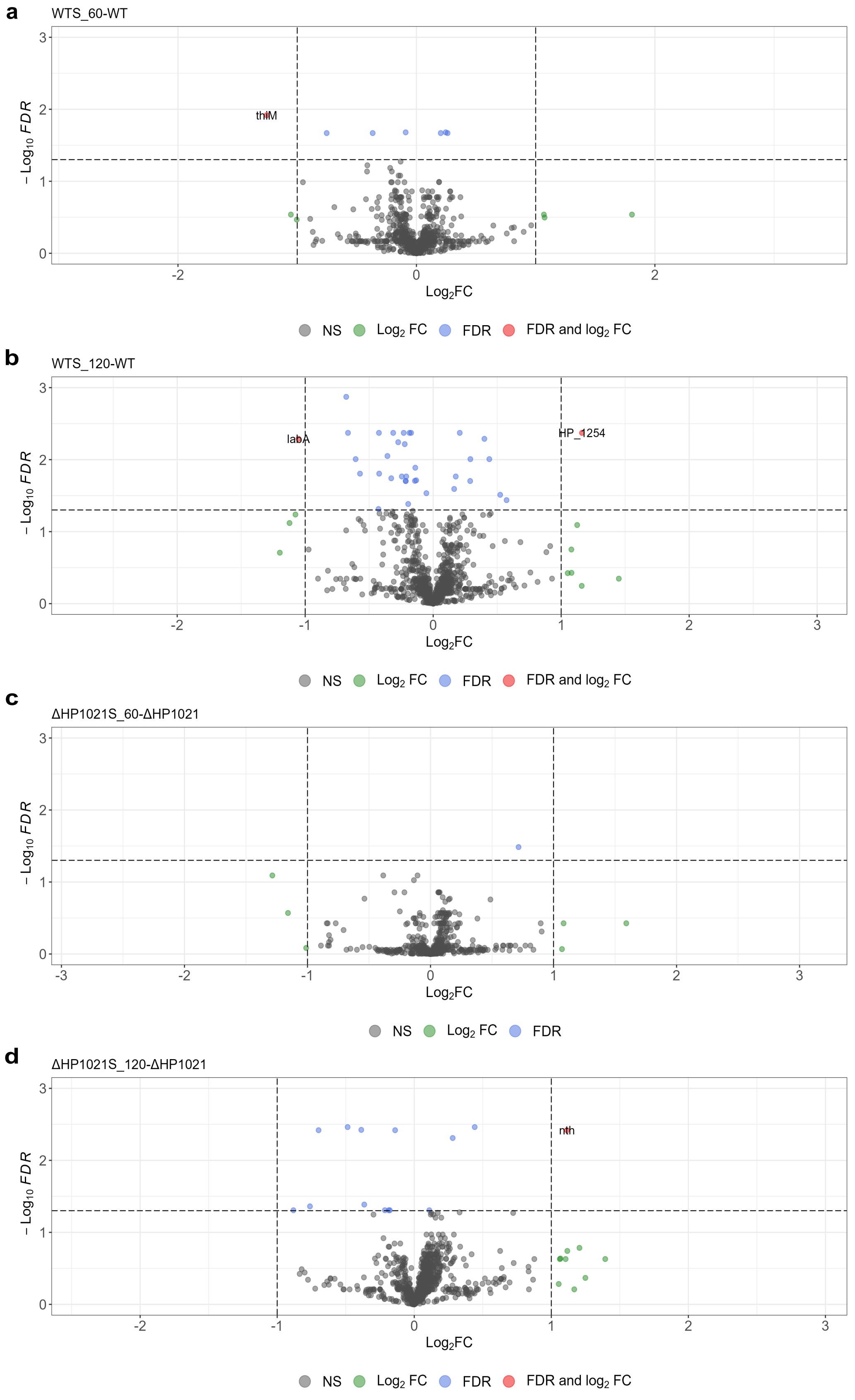

Fig. S5: Overview of the protein level regulation mediated by HP1021 revealed by MS/LC-MS. a Volcano diagram of proteins differentially expressed in the wild-type strain after 60-min oxidative stress (WTS_60) compared to the non-stressed WT strain (WTS_60-WT). **b** Volcano diagram of proteins differentially expressed in the wild-type strain after 120 min of oxidative stress (WTS_120) compared to the non-stressed wild-type strain (WTS_120-WT). **c** Volcano diagram of proteins differentially expressed in the ΔHP1021 mutant strain after 60 min of oxidative stress (ΔHP1021S_60) compared to the non-stressed ΔHP1021 mutant strain (ΔHP1021-ΔHP1021S_120). **d** Volcano diagram of proteins differentially expressed in the ΔHP1021 mutant strain after 120 min of oxidative stress (ΔHP1021S_120) compared to the non-stressed ΔHP1021 mutant strain (ΔHP1021S_120-ΔHP1021). **a-d** Four independent biological replicates were analyzed. Green dots correspond to genes with $|\log_2 FC| \geq 1$ and $FDR \geq 0.05$; blue dots correspond to genes with $|\log_2 FC| \leq 1$ and $FDR \leq 0.05$; red dots correspond to genes with $|\log_2 FC| \geq 1$ and $FDR \leq 0.05$; grey dots correspond to genes that were not significantly changed. NS, non significant. FDR, false discovery rate.

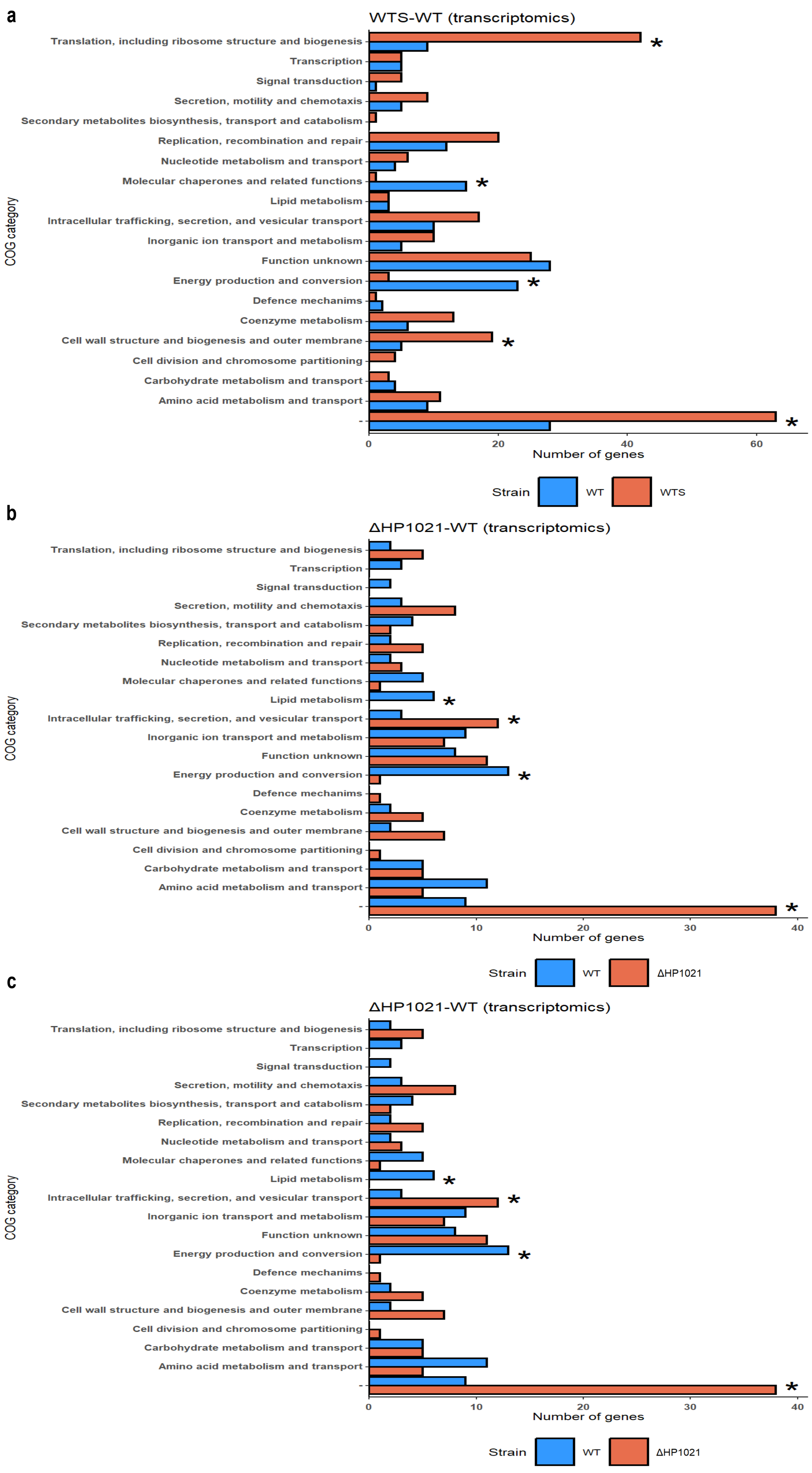

Fig. S6: *H. pylori* N6 Clusters of Orthologous Groups (COG). a COG groups of genes differently transcribed in the wild-type strain under oxidative stress (WTS) compared to the non-stressed wild-type strain (WTS-WT). **b** COGgroups of genes differently transcribed in the ΔHP1021 mutant strain compared to the wild-type (WT) strain (ΔHP1021-WT). **c** COGgroups of proteins expressed differently transcribed in the ΔHP1021 mutant strain compared to the wild-type (WT) strain (ΔHP1021-WT). **a-b** Chi-squared test determined the P value; significantly affected COGs ($P \leq 0.05$) are marked with black stars.

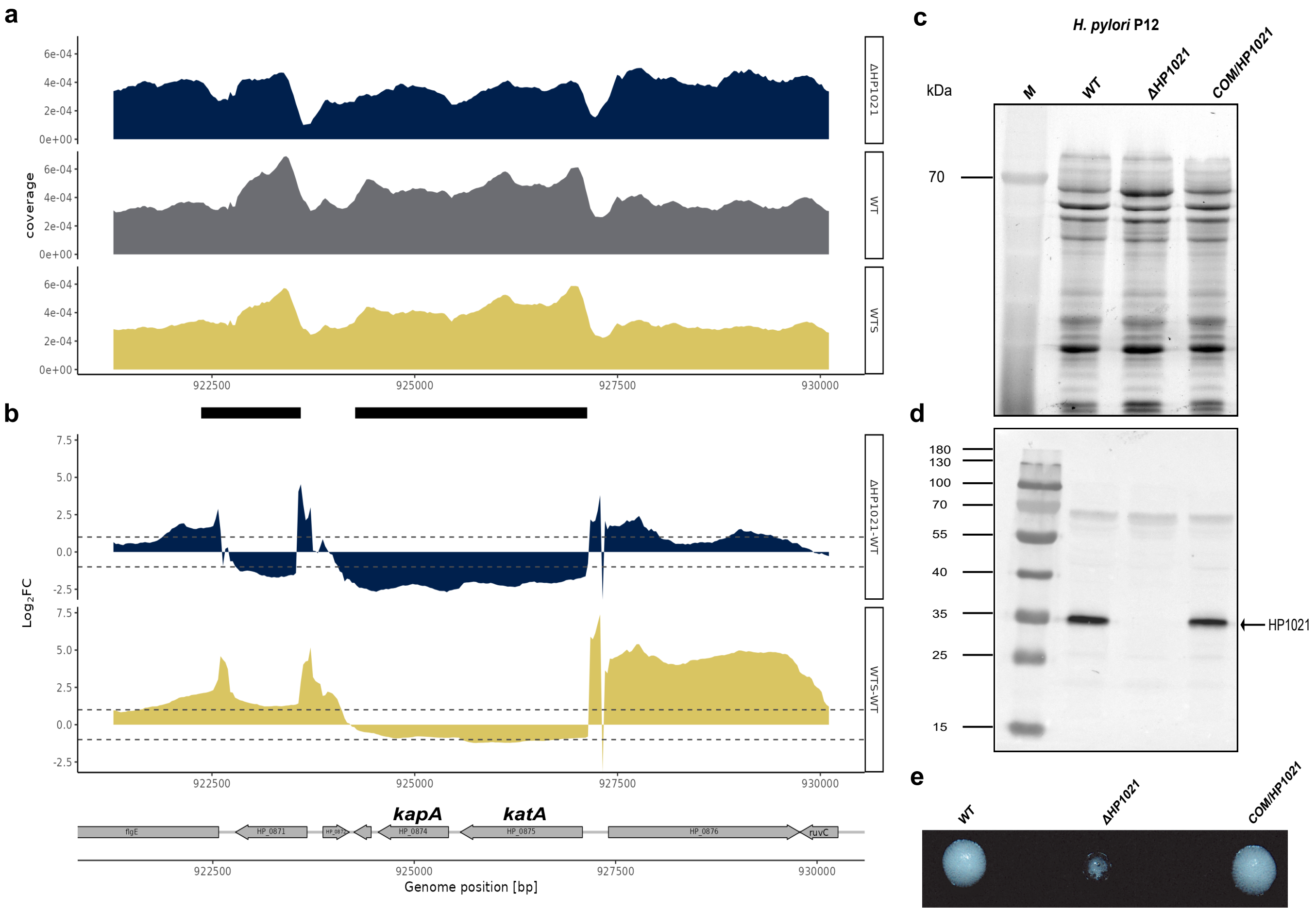

Fig. S7: HP1021 controls *katA* and *kapA* expression. a ChIP-seq data profile of the *katA* and *kapA* genes. Read counts were determined for *H. pylori* N6 WT, WTS and ΔHP1021 strains. The y-axis represents the coverage of the DNA reads, while the x-axis represents the position of the genome (in bps). The main peak of the binding site is marked with a thick black line under the x-axis. **b** RNA-seq data profile of *katA* and *kapA* genes. The genomic locus for *H. pylori* N6 WT, WTS and ΔHP1021 strains with the WTS-WT and ΔHP1021-WT expression comparison; values above the black dashed lines indicate a change in the expression of $|\log_2 FC| \geq 1$; FDR ≤ 0.05. **c** Western blot analysis of HP1021 in *H. pylori* P12 wild-type and mutant strains. Lysate of each *H. pylori* strain (approximately 1.4×10^8 cells per well) was resolved in a 10% SDS-PAGE gel visualized by the TCE-UV method. **d** HP1021 was detected in bacterial lysates by a rabbit polyclonal anti-6HisHP1021 IgG. The SDS-PAGE and Western blot were performed as previously[2]. M, PageRuler Prestained Protein Ladder (Thermo Fisher Scientific). **e** Liquid cultures (10 µl) of *H. pylori* P12 of similar cell density (OD_{600} ~1) were treated with an equal volume of 30% H_2O_2. Air bubbles produced by catalase are visible as white spots. A significant decrease in foam indicates lower catalase activity in the ΔHP1021 cells. **c-e** The experiments were repeated twice with similar results. Digital processing was applied equally across the entire image. Source data are provided with this paper.

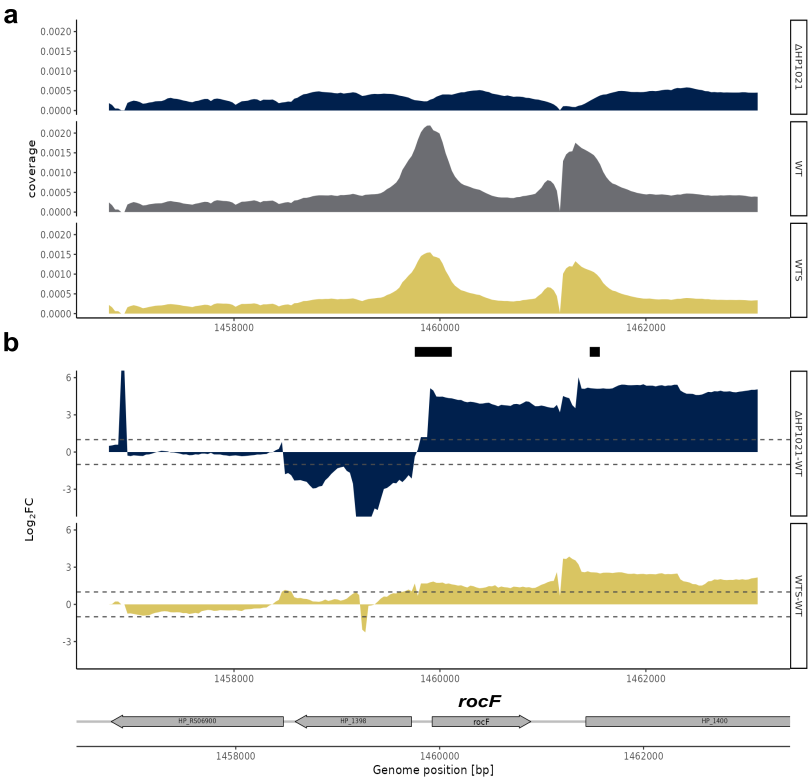

Fig. S8: HP1021 controls *rocF* expression. a ChIP-seq data profile of the *rocF* gene. Read counts were determined for *H. pylori* N6 WT, WTS and ΔHP1021 strains. The y-axis represents the coverage of the DNA reads, while the x-axis represents the position of the genome (in bps). The main peak of the binding site is marked with a thick black line under the x-axis. **b** RNA-seq data profile of *rocF* gene. The genomic locus for *H. pylori* N6 WT, WTS and ΔHP1021 strains with the WTS-WT and ΔHP1021-WT expression comparison; values above the black dashed lines indicate a change in the expression of $|\log_2 FC| \geq 1$; FDR ≤ 0.05.

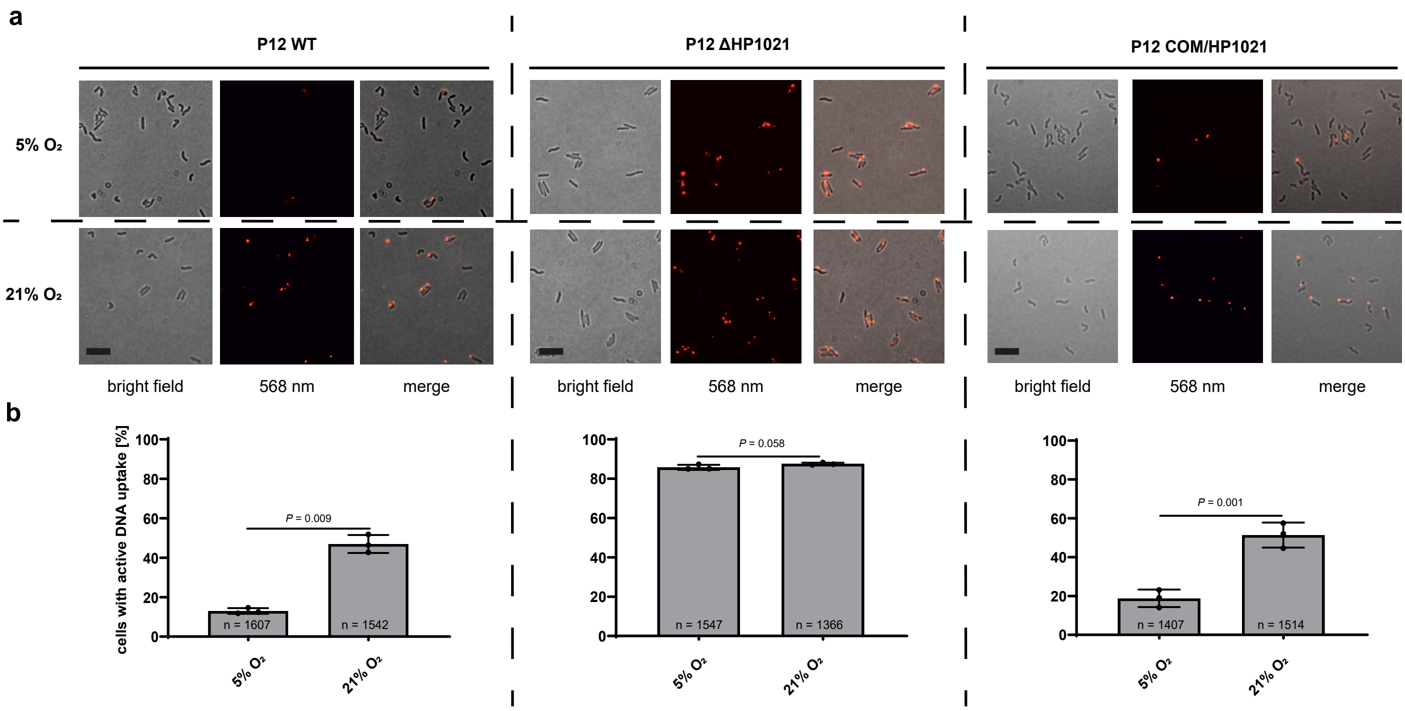

Fig. S9: Analysis of DNA uptake by *H. pylori* P12. a Bright field, fluorescent (532 nm) and merged images of *H. pylori* WT and mutant strains after 15 min of Cy3-λ DNA uptake under microaerobic and aerobic conditions (5% and 21% O_2, respectively). **b** Quantitative analysis of λ-Cy3 DNA foci formation in *H. pylori* under microaerobic and aerobic conditions (5% and 21% O_2, respectively). The scale bar represents 2 µm. Data are depicted as the mean values ± SD. Two-tailed Student's t-test determined the P value. n = number of cells examined over 3 independent biological experiments. Source data are provided with this paper.

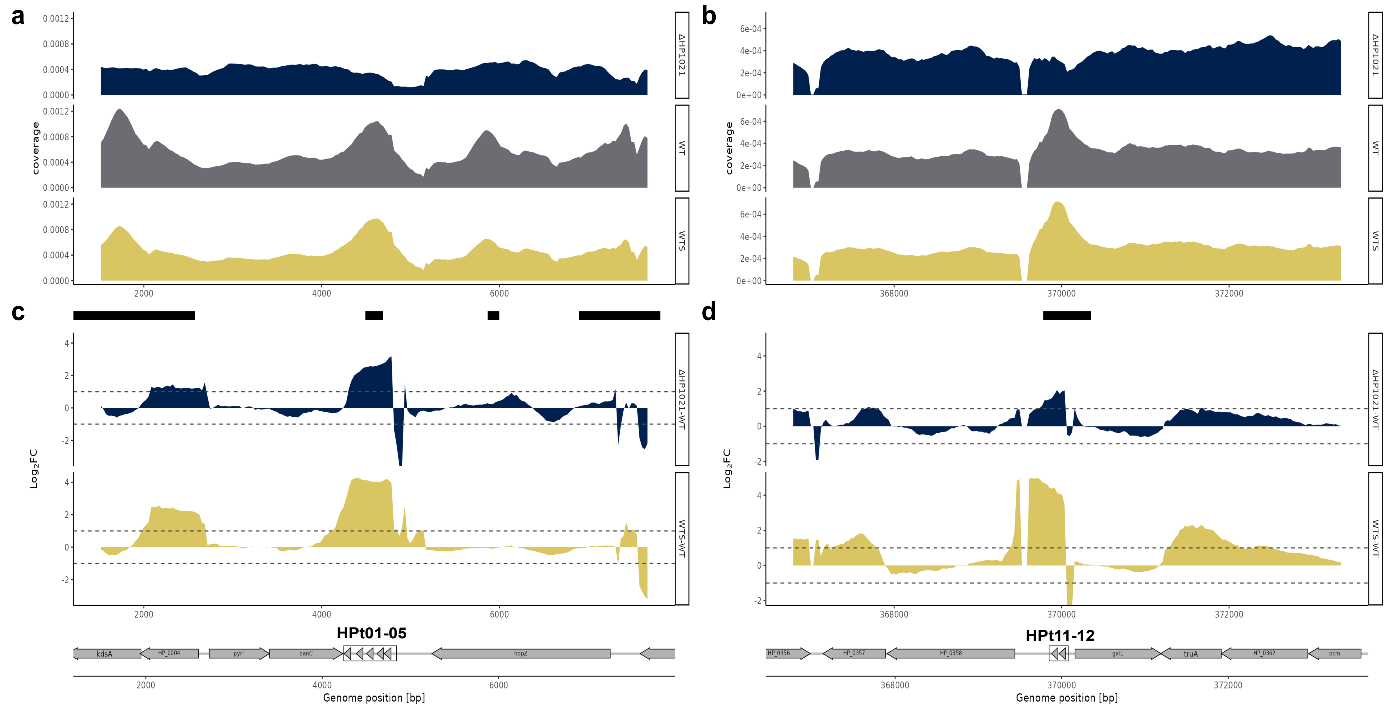

Fig. S10: HP1021 controls tRNA expression. a-b. ChIP-seq data profile of the regions coding tRNAs, namely (a) HPt01-HPt05 and (b) HPt11-HPt12. Read counts were determined for *H. pylori* N6 WT, WTS and ΔHP1021 strains. The y-axis represents the coverage of the DNA reads, while the x-axis represents the position of the genome (in bps). The main peak of the binding site is marked with a thick black line under the x-axis. **c-d** RNA-seq data profile of the regions coding tRNAs, namely (c) HPt01-HPt05 and (d) HPt11-HPt12. The genomic locus for *H. pylori* N6 WT, WTS and ΔHP1021 strains with the WTS-WT and ΔHP1021-WT expression comparison; values above the black dashed lines indicate a change in the expression of $|\log_2 FC| \geq 1$; FDR ≤ 0.05.

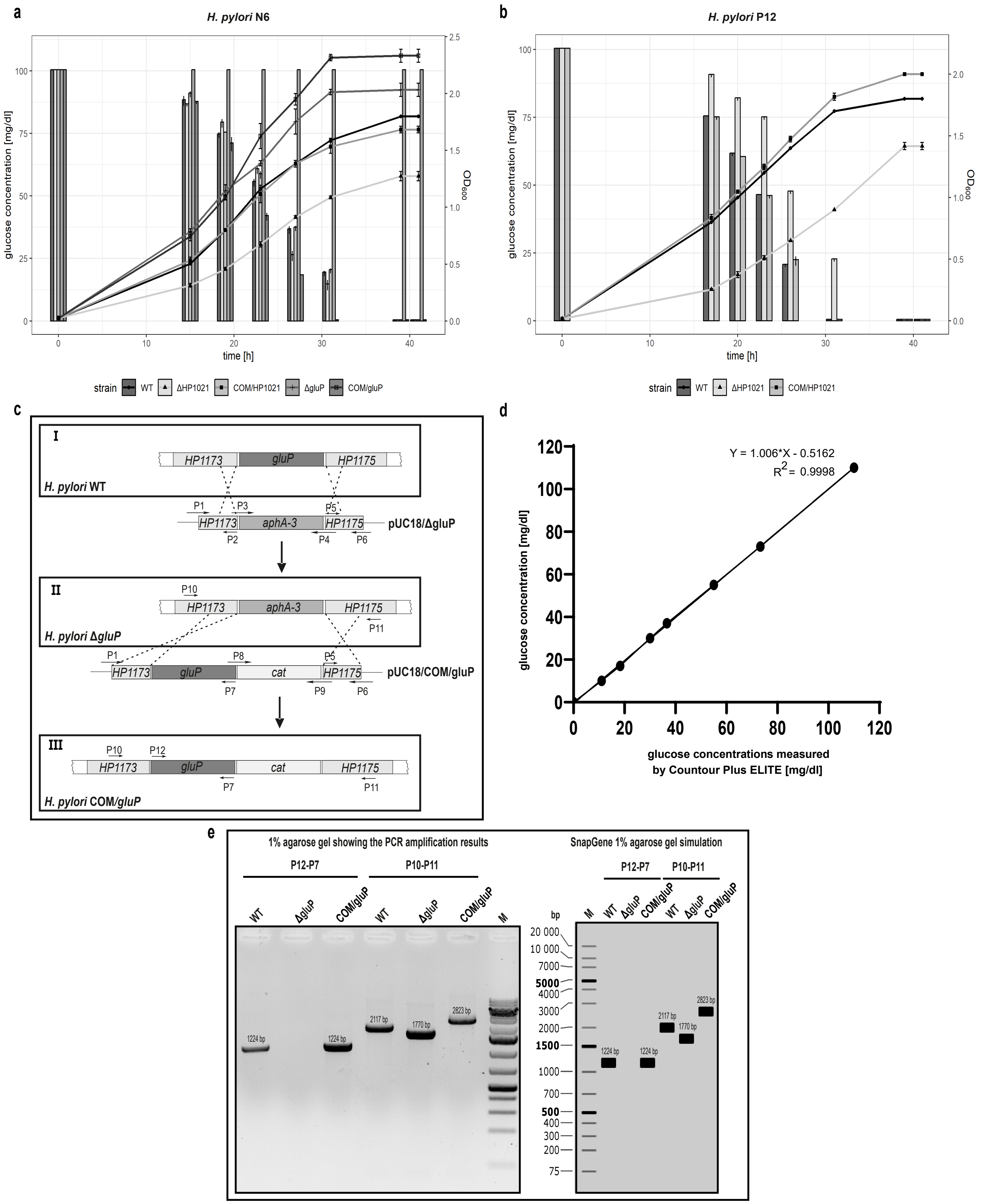

Fig. S11: HP1021 controlled glucose uptake via GluP transporter. a Growth curves of *H. pylori* N6 wild-type and mutant strains (line plot), combined with glucose concentration in the culture (bar plot). **b** Growth curves of *H. pylori* P12 WT, ΔHP1021, COM/HP1021 combined with glucose consumption (bar plot). **c** The mutagenesis strategy used to delete and subsequently complement *gluP* on the *H. pylori* N6 chromosome. *H. pylori* N6 wild-type *gluP* chromosomal loci (I) and plasmid DNA for double crossing-over to give *H. pylori* N6 ΔgluP (II) and COM/gluP (III) mutant strains are shown. For the plasmids and primer sequences, see Supplementary Tables S1 and S2, respectively. **d** Glucose concentration standard curve. The TSBΔD-FBS medium supplemented with glucose (110 mg/dl) was serially diluted with 1 × PBS, and the glucose concentration of the appropriate dilutions was measured with Contour Plus ELITE. **e** Agarose gel electrophoresis of PCR products confirming the correct *H. pylori* N6 ΔgluP and COM/gluP mutant strain construction with SnapGene® agarose gel simulation. M, GeneRuler™ 1 kb Plus DNA Ladder (Thermo Fisher Scientific). The experiment was repeated once. **a-b** n = 3 biologically independent experiments. Source data are provided with this paper.

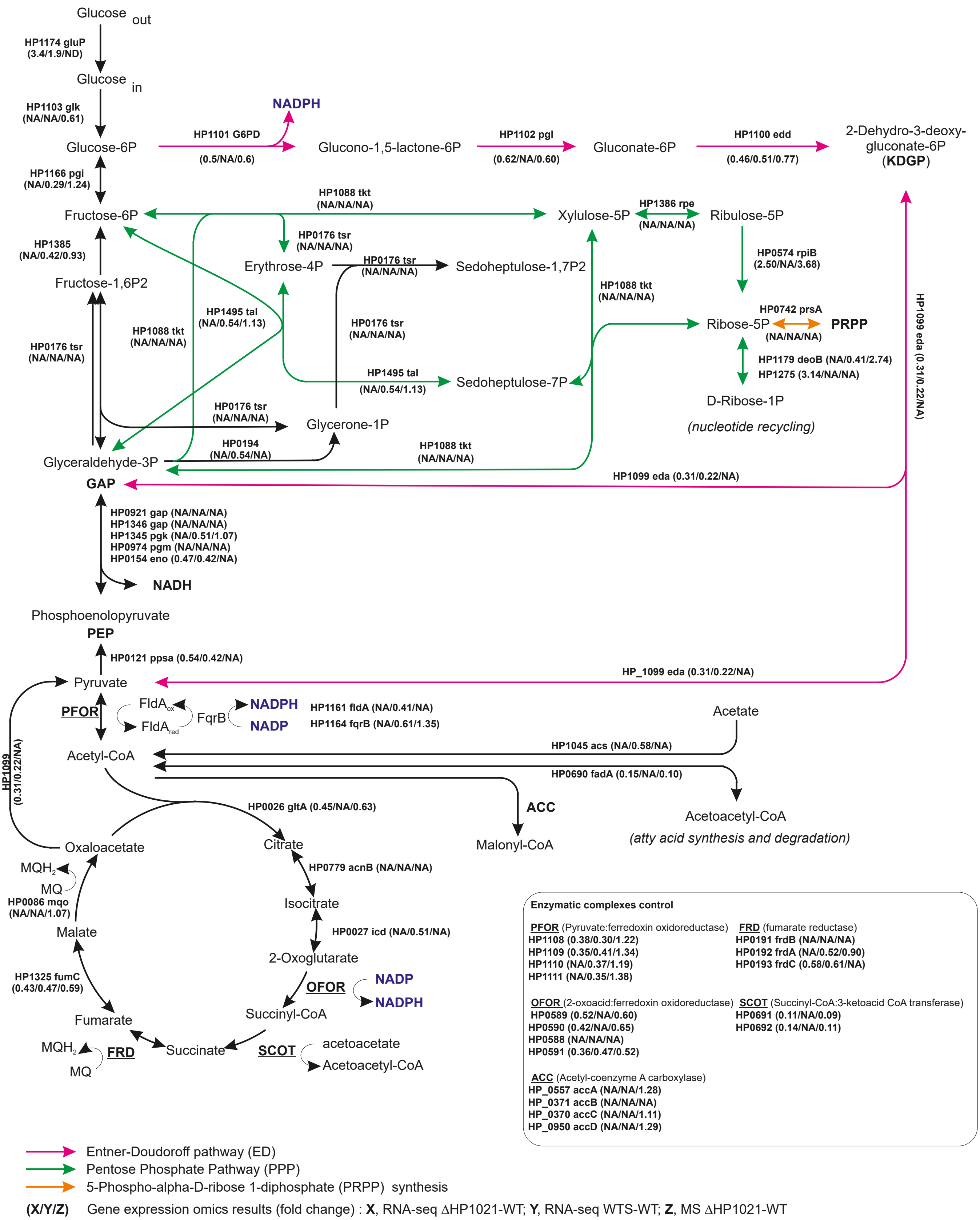

Fig. S12: A model of glucose metabolism in *H. pylori* N6 based on KEGG database and Steiner et al.[7]. Genes annotation according to *H. pylori* 26695 (NC_000915.1) strain . MQ, menaquinone; Fd, ferredoxin; NA, indifferent gene (the change was not significant); ND, not detected.

Supplementary References

1. Sambrook, J. & Russel, D. W. *Molecular Cloning: A Laboratory Manual*. *Cold Spring Harbor Laboratory Press* Cold Spring Harbor Laboratory Press, New York (2001).
2. Tomb, J. F. *et al.* The complete genome sequence of the gastric pathogen *Helicobacter pylori*. *Nature* **388**, 539–547 (1997).
3. Ferrero, R. L., Cussac, V., Courcoux, P. & Labigne, A. Construction of isogenic urease-negative mutants of *Helicobacter pylori* by allelic exchange. *J. Bacteriol.* **174**, 4212–4217 (1992).
4. Szczepanowski, P. *et al.* HP1021 is a redox switch protein identified in *Helicobacter pylori*. *Nucleic Acids Res.* **49**, 6863–6879 (2021).
5. Donczew, R. *et al.* The atypical response regulator HP1021 controls formation of the *Helicobacter pylori* replication initiation complex. *Mol. Microbiol.* **95**, 297–312 (2015).
6. Donczew, R., Weigel, C., Lurz, R., Zakrzewska-Czerwińska, J. & Zawilak-Pawlik, A. *Helicobacter pylori oriC* — the first bipartite origin of chromosome replication in Gram-negative bacteria. *Nucleic Acids Res.* **40**, 9647 (2012).
7. Steiner, T. M. *et al.* Substrate usage determines carbon flux via the citrate cycle in *Helicobacter pylori*. *Mol. Microbiol.* **116**, 841–860 (2021).
