# Supplementary material for: Profiling of the Helicobacter pylori redox switch HP1021 regulon using a multi-omics approach
Source: Nat Commun. 2023 Oct 23;14:6715. doi: 10.1038/s41467-023-42364-6 (PMC10593804; doi:10.1038/s41467-023-42364-6)
Supplement: Supplementary file 3 — Description of Additional Supplementary Files [file 41467_2023_42364_MOESM3_ESM.pdf]

## Description of Additional Supplementary Files

**Supplementary Data 1:**
Identification of HP1021 binding sites on *H. pylori* N6 genome by ChIP-seq. Chromosome position defined according to *H. pylori* 26695 strain (NC_000915.1 ).

**Supplementary Data 2:**
Analysis of HP1021 dependent gene expression in *H. pylori* N6 WT and ΔHP0121 strains under microaerobic and aerobic conditions. RNA-seq, ChIP-seq and LC-MS/MS data are presented. Genes annotation according to *H. pylori* 26695 strain (NC_000915.1).

**Supplementary Data 3:**
Comprehensive ChIP-seq, RNA-seq and MS data results for genes of selected processes or pathways in *H. pylori* N6. Genes annotation according to *H. pylori* 26695 strain (NC_000915.1). ComB uptake system according to Fisher et al., 2020 and Damke et al., 2022. The glucose metabolism pathway is based on the KEGG database, Kather at al. 2000, Corthesy-Theulaz et al. 1997 and Steiner et al. 2021. ROS and RNS response according to Flint et. al 2016. Transcriptomic and proteomic data are presented as $log_2$-fold changes. Legends specific to each pathway/process were added below particular tables. For more data, see Supplementary Data 1.
