# Supplementary material for: Profiling of the Helicobacter pylori redox switch HP1021 regulon using a multi-omics approach
Source: Nat Commun. 2023 Oct 23;14:6715. doi: 10.1038/s41467-023-42364-6 (PMC10593804; doi:10.1038/s41467-023-42364-6)
Supplement: Supplementary file 7 — Reporting Summary [file 41467_2023_42364_MOESM7_ESM.pdf]

nature portfolio

Corresponding author(s): Anna Zawilak-Pawlik

Last updated by author(s): Sep 27, 2023

Reporting Summary

Nature Portfolio wishes to improve the reproducibility of the work that we publish. This form provides structure for consistency and transparency in reporting. For further information on Nature Portfolio policies, see our Editorial Policies and the Editorial Policy Checklist.

Statistics

For all statistical analyses, confirm that the following items are present in the figure legend, table legend, main text, or Methods section.

n/a	Confirmed	
☐	☒	The exact sample size (*n*) for each experimental group/condition, given as a discrete number and unit of measurement
☐	☒	A statement on whether measurements were taken from distinct samples or whether the same sample was measured repeatedly
☐	☒	The statistical test(s) used AND whether they are one- or two-sided<br>*Only common tests should be described solely by name; describe more complex techniques in the Methods section.*
☒	☐	A description of all covariates tested
☐	☒	A description of any assumptions or corrections, such as tests of normality and adjustment for multiple comparisons
☐	☒	A full description of the statistical parameters including central tendency (e.g. means) or other basic estimates (e.g. regression coefficient) AND variation (e.g. standard deviation) or associated estimates of uncertainty (e.g. confidence intervals)
☐	☒	For null hypothesis testing, the test statistic (e.g. *F*, *t*, *r*) with confidence intervals, effect sizes, degrees of freedom and *P* value noted<br>*Give P values as exact values whenever suitable.*
☒	☐	For Bayesian analysis, information on the choice of priors and Markov chain Monte Carlo settings
☒	☐	For hierarchical and complex designs, identification of the appropriate level for tests and full reporting of outcomes
☒	☐	Estimates of effect sizes (e.g. Cohen's *d*, Pearson's *r*), indicating how they were calculated

Our web collection on statistics for biologists contains articles on many of the points above.

Software and code

Policy information about availability of computer code

Data collection	ImageQuant (v. 8.1),<br>ImageLab (v. 5.2),<br>Axiovision (v. 4.8).
Data analysis	Bowtie2 (v. 2.3.5.1),<br>samtools (v. 1.10),<br>Rsubread (v. 2.10),<br>edgeR (v. 3.38),<br>MACS3 (v.3.0)<br>csaw (v. 1.30),<br>MaxQuant (v. 1.6.3.4),<br>CFX Maestro (v. 1.1),<br>GraphPad Prism (v. 8.4.2),<br>R (v. 4.2).

For manuscripts utilizing custom algorithms or software that are central to the research but not yet described in published literature, software must be made available to editors and reviewers. We strongly encourage code deposition in a community repository (e.g. GitHub). See the Nature Portfolio guidelines for submitting code & software for further information.

Data

Policy information about availability of data

All manuscripts must include a data availability statement. This statement should provide the following information, where applicable:

- Accession codes, unique identifiers, or web links for publicly available datasets
- A description of any restrictions on data availability
- For clinical datasets or third party data, please ensure that the statement adheres to our policy

The RNA-seq FASTQ and processed data generated in this study have been deposited in the ArrayExpress database (EMBL-EBI) under accession code E-MTAB-13025 (https://www.ebi.ac.uk/biostudies/arrayexpress/studies/E-MTAB-13025?key=bf2d8677-1b26-4f1e-ac5f-b1c78c7590be).
The ChIP-seq FASTQ and BED files generated in this study have been deposited in the ArrayExpress database (EMBL-EBI) under accession code E-MTAB-13026 (https://www.ebi.ac.uk/biostudies/arrayexpress/studies/E-MTAB-13026?key=2b27b413-e118-4802-859e-5da57ff1ead2).
The raw proteomics data, MaxQuant search results, and the used protein sequence database generated in this study have been deposited in the ProteomeXchange Consortium via the PRIDE partner repository under accession code PXD041978 (http://proteomecentral.proteomexchange.org/cgi/GetDataset?ID=PXD041978).
Helicobacter pylori 26695 reference genome is deposited in the National Center for Biotechnology Infromation under accession code NC_000915.1 (www.ncbi.nlm.nih.gov/nuccore/NC_000915.1?report=genbank).
Source data are provided with this paper.

Research involving human participants, their data, or biological material

Policy information about studies with human participants or human data. See also policy information about sex, gender (identity/presentation), and sexual orientation and race, ethnicity and racism.

Reporting on sex and gender	n/a
Reporting on race, ethnicity, or other socially relevant groupings	n/a
Population characteristics	n/a
Recruitment	n/a
Ethics oversight	n/a

Note that full information on the approval of the study protocol must also be provided in the manuscript.

Field-specific reporting

Please select the one below that is the best fit for your research. If you are not sure, read the appropriate sections before making your selection.

☒ Life sciences ☐ Behavioural & social sciences ☐ Ecological, evolutionary & environmental sciences

For a reference copy of the document with all sections, see nature.com/documents/nr-reporting-summary-flat.pdf

Life sciences study design

All studies must disclose on these points even when the disclosure is negative.

Sample size	No sample size calculation was performed. RNA-seq and ChIP-seq experiments were performed with 3 biologigal replicates base on the time, cost and convenience of data collection with sufficient statistical power. The proteomics experiment was performed with four biological replicates based on the time, cost and convenience of data collection with adequate statistical power.
Data exclusions	No data were excluded from the analysis.
Replication	Biological replicates were cultures started independently from a glycerol stock on different days. The RNA-seq and ChIP-seq data were collected from 3 independent experiments. The LC-MS/MS (proteomics) data were collected from 4 independent experiments. The microscopy (Cy3-λ DNA uptake) data were collected from at least 3 independent experiments. The transformation rate glucose uptake and relative ATP production data were collected from 3 independent experiments. EMSA and catalase assay were repeated two times. Only successful experiments were regarded as replicates.
Randomization	Randomization was not relevant to this investigation. All samples were subjected to the same screening methods.
Blinding	Blinding was not relevant to this investigation. All samples were subjected to the same screening methods.

Reporting for specific materials, systems and methods

We require information from authors about some types of materials, experimental systems and methods used in many studies. Here, indicate whether each material, system or method listed is relevant to your study. If you are not sure if a list item applies to your research, read the appropriate section before selecting a response.

Materials & experimental systems

n/a	Involved in the study
☐	☒ Antibodies
☒	☐ Eukaryotic cell lines
☒	☐ Palaeontology and archaeology
☒	☐ Animals and other organisms
☒	☐ Clinical data
☒	☐ Dual use research of concern
☒	☐ Plants

Methods

n/a	Involved in the study
☐	☒ ChIP-seq
☒	☐ Flow cytometry
☒	☐ MRI-based neuroimaging

Antibodies

Antibodies used

Anti-HP1021 antibody: the immunoglobulin G (IgG) fraction of rabbit antibodies containing the anti-6HisHP1021 antibody was obtained by ammonium sulfate precipitation from rabbit serum. The antibody was prepared in our laboratory, approved by the First Local Committee for Experiments with the Use of Laboratory Animals, Wroclaw, Poland (permission number 51/2012).
Anti-rabbit antibody: rabbit IgG HRP-linked whole Ab, NA934 GE Healthcare, LOT 9494122.

Validation

The anti-HP1021 antibody was validated using extracts from bacterial strains mutated in the HP1021 gene (doi.org/10.1111/mmi.12866; Fig. 2 A).

Plants

Seed stocks

n/a

Novel plant genotypes

n/a

Authentication

n/a

ChIP-seq

Data deposition

☒ Confirm that both raw and final processed data have been deposited in a public database such as GEO.

☒ Confirm that you have deposited or provided access to graph files (e.g. BED files) for the called peaks.

Data access links
May remain private before publication.

The ChIP-seq FASTQ and BED files have been deposited in the ArrayExpress database (EMBL-EBI) and can be accessed using the dataset identifier E-MTAB-13026 (https://www.ebi.ac.uk/biostudies/arrayexpress/studies/E-MTAB-13026?key=2b27b413-e118-4802-859e- 5da57ff1ead2).

Files in database submission

D_1_1.fq.gz
D_1_2.fq.gz
D_2_1.fq.gz
D_2_2.fq.gz
D_3_1.fq.gz
D_3_2.fq.gz
D_4_1.fq.gz
D_4_2.fq.gz
D_5_1.fq.gz
D_5_2.fq.gz
D_6_1.fq.gz
D_6_2.fq.gz
R10_D_1.fq.gz
R10_D_2.fq.gz
R10S_D_1.fq.gz
R10S_D_2.fq.gz
R11_D_1.fq.gz
R11_D_2.fq.gz
R11S_D_1.fq.gz
R11S_D_2.fq.gz

R12_D_1.fq.gz
R12_D_2.fq.gz
R12S_D_1.fq.gz
R12S_D_2.fq.gz
peaks_ChIPseq_N6.bed

Genome browser session (e.g. UCSC)

n/a

Methodology

Replicates

The ChIP-seq data were collected from 3 independent experiments (biological replications).

Sequencing depth

total number / uniquely mapped reads

D1 10163703/9332312
D2 11928945/10910213
D3 13599676/12440983
D4 13713527/12351773
D5 13542187/12418185
D6 11436611/10493090
R10 11079644/11079644
R10S 10953659/10179235
R11 17236594/15700813
R11S 14374049/13442610
R12 11173477/10248313
R12S 11522928/10490473

read lenght 150 bp, paired-end

Antibodies

anti-HP1021 antibody (for more details, see Antibodies section).

Peak calling parameters

Peak calling parameters:

bowtie2 - local
edgeR
width: 100 bp
spacing: 33 bp
filter type: localbin
length for filter: 2000 bp
minimal quality: 30
used glmQLFTest function with mergeWindows (merge distance = 100 bp, maximum peak length = 5000 bp), combineTests and getBestTest function

control files:
D1 D2 D3 to R10 R11 R12

Data quality

Read files were checked using fastqc.
ChIP-seq analysis followed established protocol. Peaks with logFC < 2 were removed from further analysis.
edgeR functions: combineTests and getBestTest were used to adjust for multiple comparisons.
Identified peaks were independently confirmed using MACS3 program.
edgeR: Peaks below FDR < 0.05 and logFC > 2 (all found peaks): WT vs ΔHP1021 56 (48) MACS3 WT vs ΔHP1021 82 (61).

Software

Data analysis reads were mapped to the H. pylori 26695 genome (NC_000915.1) using Bowtie2 (v. 2.3.5.1) and processed using samtools (v. 1.10). Regions differentially bound by HP1021 were identified using MACS3 (version 3.0.0a6) and R packages csaw (v. 1.30), and edgeR (v. 3.38).
